# Open Your Windows

**Published:** 1874-12

**Authors:** 


Open your Windows.—There is good sense in this. There are some houses in every town whose windows might as well be sealed in with the walls for any purpose they have but to let in the light. They are never opened, summer or winter. In winter it is cold; in summer the flies stray in, or, if they are netted, the dust sifts through the nets. Now, you can tell a person who inhabits such chambers when you pass him in the street—there is such a smell about his clothing. You long for a sniff of cologne or hartshorn, or burnt feathers, or something of the sort to "take the taste out." A house that is never aired has every nook and corner filled with stale odors of cooked meats, boiled vegetables, especially cabbage and onions, which, as the weeks go by, literally reek in their hiding places. Who has not wished sometimes to hang a new servant's clothing out of doors some frosty night until it should be thoroughly aired ? Fine ladies come sweeping into church with their velvets and silks, said velvets and silks giving unmistakable evidence of having been housed in just such shut-up chambers. Oh, what a tale that odor of pork and cabbage tells about the lady's style of housekeeping! The very garments of the children tell the same story of uncleanliness. It is bad to have unwashed clothes, but there may be an excuse for it. But what excuse can there be for unaired ones, when the air is so cheap and free? There is death in such close rooms. Better a swarm of flies or a cloud of dust; better frost and snow in a room than these intolerable smells. Dear girls, the first thing in the morning, when you are ready to go down stairs, throw open your windows, take apart the clothing of your beds, and let the air blow through as hard as it will. There is health and wealth in such a policy. It helps to keep away the doctors with long bills. It helps to make your eyes sparkle and to make your cheeks glow, and to make others love your presence. Girls who live in those close, shut-up rooms can barely be tolerated, at the best, in any circle.

